# Novel image analysis tool for rapid screening of cell morphology in preclinical animal models of disease

**DOI:** 10.1016/j.heliyon.2023.e13449

**Published:** 2023-02-08

**Authors:** Michelle Guignet, Martin Schmuck, Danielle J. Harvey, Danh Nguyen, Donald Bruun, Angela Echeverri, Gene Gurkoff, Pamela J. Lein

**Affiliations:** a Department of Molecular Biosciences, School of Veterinary Medicine, University of California-Davis, 1089 Veterinary Medicine Drive, Davis, CA, 95616, USA; b Department of Public Health Sciences, University of California-Davis, One Shields Avenue, Davis, CA, 95616, USA; c Division of General Internal Medicine, Department of Medicine, School of Medicine, University of California-Irvine, 100 Theory, Suite 120, Irvine, CA, 92617, USA; d Department of Neurological Surgery, School of Medicine, University of California-Davis, 4800 Y Street, Sacramento, CA, 95817, USA; e Center for Neuroscience, University of California-Davis, 1544 Newton Court, Davis, CA, 95618, USA; f MIND Institute, School of Medicine, University of California-Davis, 2825 50th Street, Sacramento, CA, 95817, USA

**Keywords:** Automated image analysis, Microglia, Morphology, Organophosphate, Traumatic brain injury, Unbiased image analysis

## Abstract

The field of cell biology has seen major advances in both cellular imaging modalities and the development of automated image analysis platforms that increase rigor, reproducibility, and throughput for large imaging data sets. However, there remains a need for tools that provide accurate morphometric analysis of single cells with complex, dynamic cytoarchitecture in a high-throughput and unbiased manner. We developed a fully automated image-analysis algorithm to rapidly detect and quantify changes in cellular morphology using microglia cells, an innate immune cell within the central nervous system, as representative of cells that exhibit dynamic and complex cytoarchitectural changes. We used two preclinical animal models that exhibit robust changes in microglia morphology: (1) a rat model of acute organophosphate intoxication, which was used to generate fluorescently labeled images for algorithm development; and (2) a rat model of traumatic brain injury, which was used to validate the algorithm using cells labeled using chromogenic detection methods. All *ex vivo* brain sections were immunolabeled for IBA-1 using fluorescence or diaminobenzidine (DAB) labeling, images were acquired using a high content imaging system and analyzed using a custom-built algorithm. The exploratory data set revealed eight statistically significant and quantitative morphometric parameters that distinguished between phenotypically distinct groups of microglia. Manual validation of single-cell morphology was strongly correlated with the automated analysis and was further supported by a comparison with traditional stereology methods. Existing image analysis pipelines rely on high-resolution images of individual cells, which limits sample size and is subject to selection bias. However, our fully automated method integrates quantification of morphology and fluorescent/chromogenic signals in images from multiple brain regions acquired using high-content imaging. In summary, our free, customizable image analysis tool provides a high-throughput, unbiased method for accurately detecting and quantifying morphological changes in cells with complex morphologies.

## Abbreviations

AS: atropine sulfate
CD68: cluster of differentiation 68
CNS: central nervous system
DFP: diisopropylfluorophosphate
IBA-1: ionized calcium binding adaptor molecule 1
NIH: National Institutes of Health
OP: organophosphate
PB: phosphate buffer
PBS: phosphate-buffered saline
ROI: region of interest
SD: standard deviation
SE: status epilepticus
SEM: standard error
TBI: traumatic brain injury
VEH: vehicle

## 1 Introduction

Significant advances in the development of imaging modalities over the past few decades have improved scientists’ ability to monitor biological phenomena with extraordinary resolution, specificity, complexity, and scale [See Ref. [1] for review]. However, analyzing large and increasingly complex datasets is often a tedious process that creates productivity bottlenecks for many researchers, and is often susceptible to human error and investigator bias. As such, there has been a push to develop automated and unbiased image segmentation and analysis pipelines that balance rigor & reproducibility with increased-throughput and can be applied across *in vitro, ex vivo,* and *in vivo* platforms [[2], [3], [4], [5], [6], [7]].

Even with the development of various image analysis pipelines, there remains a paucity of accurate and reliable methods for analyzing cell types with more complex and dynamic cytoarchitecture, such as many cells within the central nervous system (CNS), including neurons, astrocytes, microglia, and oligodendrocytes [8]. Oftentimes, the morphologies of these cells change to support their specialized functions during health and disease. For instance, under physiologic conditions, microglia are critically important for basic homeostatic functions and general immune surveillance within the CNS [[9], [10], [11], [12]]. This is reflected by their “surveilling” morphology with a small cell soma and long, ramified processes to monitor their microenvironment [13]. In response to damage, injury, or infection, microglia condense their processes and become more amoeboid in shape to reflect a change in activation status towards a more phagocytic phenotype [[14], [15], [16]]. This change in morphology corresponds with a shift in their role towards coordinating adaptive immune responses within the brain to limit the spread of injury by phagocytosing cellular debris and promoting wound healing. In addition to morphological changes, activated microglia increase transcriptional and translational production of inflammatory cytokines and chemokines [17,18] to coordinate an inflammatory response [[19], [20], [21], [22]], facilitate migration to sites of injury [23], increase phagocytic efficiency [24] and, ultimately, benefit injured tissue by scavenging neurotoxic molecules and removing cellular debris or dying cells. Historically, microglia activation has been described in either binary or categorical terms, with M1 being “pro-inflammatory” and M2 as “anti-inflammatory” [25]; however, this classification is recognized as an oversimplification as evidence increasingly supports a model in which microglial activation is a dynamic process across a continuum [[26], [27], [28]]. For instance, until microglia become phagocytes, there is a range in the number, length, and branching complexity of cell processes that cannot be fully described using only binary or categorical terms. These diverse and dynamic phenotypes make it difficult to evaluate the status of microglial activation in the brain and represent a challenge that extend to other cell types with dynamic and complex cytoarchitecture. Ultimately, developing sensitive and accurate tools to quantify these changes across a continuum may provide better insight into the mechanisms of various cell-types in both health and disease. Existing methods for evaluating complex cell morphologies, like those of microglia, have often relied on manual scoring, which results in subjective and inherently biased data [29,30]. However, other groups have recognized the need for unbiased approaches and as such, have developed standardized methods, such as quantifying 2D (surface area) & 3D (volumetric) characteristics [[31], [32], [33]], measuring total length and branching complexity of microglial processes [34,35], or clustering microglia into distinct phenotypic subtypes based on expression of various biomarkers [36,37]. The major limitations with these approaches, however, is that some are only partially automated and rely on manual cell selection and/or tracing [29,30], they require high magnification & high-resolution images, which results in low-to medium-throughput analyses [32,34,36], and most studies report either fluorescent [35,36] or chromogen [38] detection methods, but no published method demonstrates the versatility of their tool to analyze morphologies labeled using both detection methods. There remains an unmet need to develop unbiased and automated analysis platforms that can rapidly screen large numbers of cells with the flexibility of using multiple cellular labeling methods.

Here, we describe a custom-built, fully automated algorithm that quantifies a continuum of complex cellular morphologies in a high-throughput and unbiased manner using images generated by high content imaging, allowing for the analysis of thousands of cells across multiple brain regions. Besides generating morphologic descriptors of microglia, this algorithm measures the colocalization of biological markers of activation, e.g., CD68, within individual microglia, a critical metric missing from other published automated methods [34,38]. This method was validated in two established preclinical models characterized by robust changes in microglial morphology – acute intoxication with the organophosphate cholinesterase inhibitor diisopropylfluorophosphate (DFP) [39,40] and traumatic brain injury (TBI) [41,42] – using two methods for visualizing microglia cells in brain sections – indirect immunofluorescence and chromogenic methods, respectively. While validated across a continuum of microglia phenotypes, this system can be adapted and optimized to evaluate different cell-types with complex morphologies. Our data suggest that this free, automated image analysis tool offers a novel and customizable approach for investigators to rapidly screen for morphological changes in large populations of cells in an unbiased manner. We believe this tool will advance the field of cell biology to help answer fundamental questions related to the function(s) of morphologically distinct subsets of cells in both health and disease.

## 2 Materials and methods

### 2.1 Animals and experimental models of microglial activation

Experiments involving animals complied with ARRIVE guidelines and were performed in accordance with the National Institutes of Health guide for the care and use of laboratory animals (NIH publication No. 8023, revised 1978) following protocols approved by the University of California, Davis, Institutional Animal Care and Use Committee. Adult male Sprague Dawley rats (225–250 g, 8–10 weeks old) were purchased from Charles River Laboratories and individually housed in standard plastic cages under controlled environmental conditions (22 ± 2 °C, 40–50% humidity) in fully accredited AALAC International facilities. Animals were housed in a 12 h light-dark cycle with access to food and water provided *ad libitum.* Animals were acutely intoxicated with DFP (n = 10) as previously described [43]. Vehicle (VEH, n = 15) controls were administered saline instead of DFP but were otherwise similarly treated. Experimental TBI was produced using a fluid percussion device (VCU Biomedical Engineering [44]) with the lateral orientation for impact [45] as previously published (n = 5 [46]). Sham controls (n = 5) underwent the same surgical procedures but were not administered the percussive impact. All animal and group characteristics for each data set can be found in Table 1.

**Table 1:** Animal distribution and group characteristics for exploratory and validation data sets.

	Exploratory Data Set (Algorithm Development)		Validation Data Sets			
**Detection Method**	Fluorescence		Fluorescence		Chromogen	
**Experimental Model**	DFP Intoxication		DFP Intoxication		TBI	
**Brain Region Analyzed**	Dorsal Lateral Thalamus		Dorsal Lateral Thalamus		Hippocampus	
**Final Group Size** *DFP vs. VEH* *TBI vs. Sham*	DFP: 10	VEH: 15	DFP: 10	VEH: 11	TBI: 5	Sham: 5
**Primary Antibody** *Source* *RRID*^a^	**IBA-1** *Wako Laboratory Chemicals* *AB_839504*		**IBA-1** *Wako Laboratory Chemicals* *AB_839504*		**IBA-1** *Wako Laboratory Chemicals* *AB_839504*	
	**CD68** *Bio-Rad Laboratories* *AB_2291300*		**CD68** *Bio-Rad Laboratories* *AB_2291300*			

a RRID: Research Resource Identification Number.

### 2.2 Tissue processing and immunolabeling

At 1 week following DFP or VEH exposure, animals were anesthetized with 5% isoflurane in medical grade oxygen then euthanized by exsanguination via perfusion with cold phosphate buffered saline, and their brains harvested and processed for immunohistochemical assessment of microglial morphology as previously described [47]. Brains were removed from the skull, blocked in 2-mm thick coronal sections and post-fixed for 24 h at 4 °C in 4% paraformaldehyde (PFA; Sigma-Aldrich, St. Louis, MO) in phosphate buffer (0.1 M Na_2_HPO_4_, 0.1 M NaH_2_PO_4_, pH 7.2). Tissues were then transferred to 30% (w/v) sucrose (Sigma-Aldrich) in PBS and stored at 4 °C for at least 48 h until embedded and flash frozen in Tissue-Plus™ O·C.T. compound (Thermo Fisher Scientific, Waltham, MA, USA). Tissue blocks were cryosectioned into 10 μm thick coronal sections and stored at −80 °C until immunostained. All brain sections processed for IBA-1 and CD68 labeling were immunostained at the exact same time using the same batch of reagents to ensure reproducible experimental conditions for all samples. Slides were removed from −80 °C and brought to room temperature before heating in 10 mM sodium citrate buffer (in double distilled water) at pH 6.0 and heated for 30 min in a rice cooker (Black & Decker HS2000) for antigen retrieval. After cooling to room temp, sections were incubated on slides in a blocking buffer containing 10% normal goat serum (Vector Laboratories, Burlingame, CA, USA), 1% bovine serum albumin (Sigma-Aldrich) and 0.3% Triton X-100 (PBS-T, Thermo Fisher Scientific) in PBS, for 1 h at room temperature and then incubated with primary antibody in blocking buffer at 4 °C overnight. Primary antibodies included rabbit *anti*-IBA1 (1:1000, 019–19741, Wako Laboratory Chemicals) and mouse anti-CD68 (1:200, MCA341R, Bio-Rad). Secondary antibodies included goat-anti rabbit IgG conjugated to Alexa Fluor 568 nm (1:500, A11036, Thermo Fisher Scientific, RRID: AB_10563566) to detect *anti*-IBA-1 and goat-anti mouse IgG conjugated to Alexa Fluor 488 nm (1:500, A11001, Thermo Fisher Scientific, RRID: AB_2534069) to detect anti-CD68. Negative control sections were incubated with blocking buffer instead of primary antibody. All sections were mounted in ProLong™ Gold Antifade Mountant with DAPI (Invitrogen).

Fourteen days post-TBI, rats were transcardially perfused and tissues were processed and stained as previously described [48]. Rats were euthanized by sodium pentobarbital anesthesia (100 mg/kg, ip), followed by transcardial perfusion with 100 mL of 0.1 M phosphate buffer (PB; pH = 7.4), and 350 mL of 4% paraformaldehyde (pH 7.4). Brains were extracted and post-fixed in 4% PFA at 4 °C for 1 h before moving to a 10% sucrose solution (w/v) in 0.1 M PB for 24 h, and then a 30% sucrose solution (w/v) in 0.1 M PB for 48 h and frozen at −80 °C. Tissues were mounted on a metal block and 45 μm coronal sections were cut using a sliding microtome. Every serial section starting at 1.6 mm bregma and ending at −6.3 mm bregma was saved in 0.1 M PB with sodium azide in 12-well cell culture plates and stored at 4 °C. Free floating brain sections (every 5th section spanning the rostral-caudal axis of the hippocampus) were removed from PB with azide and washed 3 × 10 min in PB before incubated at room temperature in 0.2% sodium borohydride (w/v) in PB for 15 min on a shaker, and then 15 min in 0.5% H_2_O_2_ in PB to quench endogenous peroxidase. After 3 × 10 min rinses in PB and 3 × 5 min washes in 0.01 M PBS (0.9% saline, pH 7.4), sections were incubated in 10% goat blocking serum (0.3% Triton-X in PBS) and transferred into primary antibody (Wako 019–19741; IBA-1, 1:3000 in 10% goat serum, 0.3% Triton-X and PBS) at 4 °C overnight. Tissues were incubated in a biotinylated secondary antibody (goat anti-rabbit 1:2000 in 10% goat serum, 0.3% Triton-X and PBS) for 1 h at room temperature and then placed in Vectastain ABC reagent (in 10% goat serum, 0.3% Triton-X and PBS; Vector Labs, Burlingame, CA) for 1 h at room temperature. Tissue was rinsed 3 × 5 min in PBS and then placed in chromogen (160 μL of H_2_O_2_ in PBS; Vector Labs, Burlingame, CA) and incubated for up to 10 min until desired staining was achieved.

All fluorescent and chromogen immunohistochemistry protocols were manually validated for each arm of the study and have no bearing on detection or imaging of microglia. Multiple detection methods were used to determine whether this novel image analysis tool is sensitive enough to capture changes in microglia morphology across different tissue processing and staining protocols.

### 2.3 High content imaging

Fluorescent images were acquired from the dorsal lateral thalamus of DFP and VEH animals; transmitted light images were taken from the dorsal hippocampus of TBI and sham animals. All images were acquired using ImageXpress Micro XLS Widefield High-Content Analysis System with an S Plan Fluor ELWD 20× objective. Positive immunostaining was identified as fluorescence intensity that was twice the background fluorescence levels in negative control samples and is consistent with previously published methods for the same staining protocols in strain, sex, and age-matched rats [47]. *A priori* selection of positive immunofluorescence was a preliminary step in the data analysis pipeline, explanation of additional detection and quantification methods are described in the image segmentation criteria below. Approximately 10 μm Z-stacks were acquired at optical slice intervals of 0.5 μm and collapsed into a maximum intensity projection for the final image. Each image was acquired as a 3 × 3 array of overlapping tiles that were stitched together to create a single image that encompassed the entire dorsal lateral thalamus or CA3 subregion hippocampus (5832 × 5832 pixels, resolution: 3.34 μm/pixel). Two sections were acquired from the dorsal lateral thalamus (−2.7 mm, −3.7 mm bregma) and the dorsal hippocampus (−2.5 mm, −4.5 mm bregma) for each animal, and these images were uploaded into the analysis platform.

### 2.4 Image processing and automated analysis pipeline

MATLAB scripts used for analysis, statistics and visualization can be found under https://github.com/Lein-Lab/microglia-phenotyping. The automated algorithm supports chromogenic and fluorescent images obtained from the ImageXpress Micro XL high content imaging system. Images are binarized, applying a strong and weak threshold in conjunction with edge detection and morphological closing. Only structures containing pixels from the hard threshold are considered to be valid glia structures. In the next step, the cell somata are identified by applying morphological thinning followed by opening. Thus, only the thicker cell parts remain, which correspond to the cell somata. For fluorescent images, resulting structures are overlapped with the nucleus channel. This step prevents identification of cell processes as cell bodies from microglia from other layers. Centroids of overlapping nuclei are identified and saved in a nucleus position matrix. Images of all fluorescence channels as well as their respective binary mask for cell nuclei and IBA-1 immunoreactivity are saved in the native solution and as 10% sized images for visualization in the graphic user interface. In the next step, all images are uploaded into a container within the algorithm from which they can be individually selected via an image list. Additional channels (nuclei, IBA-1 and if applicable CD68) can be shown separately or as an overlap. With the polygon filter tool, the user can select ROI's within the images. Resulting polygons are saved for later analysis.

The MeasureMicroGliaActivation function can be run in two different modes: initial analysis and validation mode. The user can choose between these two methods via a pop-up window. The initial analysis mode will analyze every single cell within a user-defined image. For the validation mode, a pre-defined number of cells are randomly selected from the image and output as single cell images for investigators to validate at a later step. All other image-processing steps remain the same between the two methods. In the first step, raw binary images from the IBA-1 channel are corrected by filling small morphological gaps and by deleting small structures. Additional objects connected by a single pixel are then separated. Next, resulting particles are overlapped with the nuclei matrix and sorted into single cells (one nucleus) or cell clusters. Consequently, the following parameters are assessed for each structure: 1) percent area, which describes the ratio of the area of the smallest bounding box around the object and the object area; 2) area of cell body (this is obtained by morphological thinning, followed by opening and consequent dilation of the remaining parts to eliminate thin structures attributed to cell processes); 3) process length; 4) morphology ratio, which is the ratio of process length and cell body area; 5) number of cells; 6) area of cell, 7) the sum intensity of CD68 (if applicable) within the binary mask of the IBA-1 channel; 8) number of endpoints of cell processes; 9) number of branching points of cell processes; 10) span ratio, which describes the ratio of the major to minor cell axis; and 11) perimeter of the cell. All results are saved in different tables for single cells, cluster and a combination of both. Utilizing the ‘Ratio of Process Length and Cell body Area’ to describe microglia activation (the smaller the ratio the more activated), cells were also grouped into a histogram for single cells, cell clusters and all cells. Furthermore, a binary image of all analyzed cells with cell body and skeletons of identified processes is saved that can be overlaid in the graphical user interface to assess quality of the analysis. In the validation mode, additional images are saved based on the degree of activation: activated cells are displayed in red, intermediate as green and non-activated cells as blue. Cropped images are saved separately allowing the experimenter to judge the scoring and to perform a manual comparison. In the final step, analysis results are recorded in an Excel spread sheet, with a separate tab for single cells, cluster, combination of cell lists and histograms.

### 2.5 Exploratory and validation data sets for automated and unbiased fluorescent detection of microglial morphology

The automated algorithm was created using fluorescent photomicrographs from the dorsal lateral thalamus in VEH and DFP animals. A total of 25 animals (VEH: 15; DFP: 10) were used to generate the algorithm in an exploratory data set: 18 animals (VEH: 8; DFP: 10) for an initial blinded and unbiased development of the algorithm and 7 VEH animals were later added as an unblinded control. The cursory review of the initial 18 animals was performed by investigators blinded to experimental groups and animal IDs. The images in the data set were separated into two distinct groups based on visual inspection of cell populations within a user-defined ROI: 1) activated microglia with the majority of IBA-1 immunopositive cells in an amoeboid state; 2) non-activated microglia with IBA-1 immunopositive cells in a predominantly ramified morphologic state. Importantly, the terms “activated” and “non-activated” were used only to describe the morphology of microglia, no further analysis or conclusions were made about activation status of these cells. Each group of images were fed through the analysis pipeline to identify specific morphometric features that separated “activated” vs. “non-activated” cell-types. An additional 7 VEH animals were then added to the analysis pipeline to serve as an unblinded control for microglia in resting morphological states. All 25 animals were used for statistical analysis (see below) to identify significant morphometric parameters of microglial activation via automated analysis.

A parallel review of the exploratory data set was performed by a different investigator (blinded to treatment groups) to manually analyze microglia counts and confirm using ImageJ v 1.52. ROIs were selected for an entire brain region and manual counts of DAPI-expressing IBA-1 cells were performed by a blinded investigator. All manual counts were confirmed with a secondary review using established ImageJ protocols that were performed as follows. Following a rolling ball background subtraction and binarization of both DAPI and IBA-1 images, the two wavelengths were combined to generate a final image with only IBA-1 cells that expressed DAPI-stained nuclei. The “analyze particles” algorithm was used to determine the number of remaining cells that were at least 10 μm in size. Manual and ImageJ counts were within 5% of each other and data are presented as the number of IBA-1 cells per unit area (mm^2^) for each animal.

The algorithm was validated in a subset of 21 animals (DFP: 10; VEH: 11) using the validation mode in the MeasureMicroGliaActivation function. Following image preprocessing, a random sample of 11-94 cells were chosen from each image and isolated into pictures of individual cells. Two brain sections were analyzed for each animal resulting in a total of 25-167 cells analyzed for each animal and a total of 1503 cells in the data set. An investigator blinded to the experimental groups scored individual cells based on a semi-quantitative scale: 1) small cell body with long, thin processes extending away from the cell body; 2) large cell body with fewer thin processes; 3) large cell body with short, thick, bushy processes; 4) completely amoeboid or rod-shaped microglia. The investigator repeated the review process three separate times (blinded to all identifying information), and the average score (rounded to the closest whole number) was compiled for each cell. After manual scoring, the investigator was unblinded to the treatment groups to compare microglial morphology between DFP and VEH groups. These data are normalized as a percentage of the total IBA-1 cells with a specific morphological score for each animal; normalized values are averaged across all animals for each treatment group. Furthermore, the accuracy of the automated system was determined based on whether the algorithm correctly defined and traced the cell body & processes for individual cells, and whether the automated morphology ratio corresponded with the semi-quantitative score assigned by a blinded investigator.

### 2.6 Stereological vs. automated assessment of microglial activation via chromogenic methods in the hippocampus following TBI

All stereology was performed by an investigator blinded to group. Tissue was examined on a microscope with a motorized stage using computer software (Stereo Investigator™ 2019 1.2; Microbrightfield). Every fifth section (ssf = 0.2) between bregma ∼ -2.56 mm to bregma ∼ -4.52 mm of the dorsal hippocampus was analyzed. ROIs were drawn using a 10× objective (Plan Apo, NA 0.45). CA3 was operationally defined by drawing a line connecting the two blades of the dentate gyrus and extending out to the pyramidal layer of the hippocampus. Pyramidal cells extending past the dentate gyrus and until the line intersects again with the pyramidal layer were defined as CA3. Cell counting was performed with a 100× oil immersion objective (Plan Apo, NA 0.95, Nikon). The total number of microglia was quantified using the optical fractionator stereological method [49]. The grid size was 100 μm^2^ with the counting frame set to 50 μm^2^ (asf of 0.25). The average section mounted thickness was ∼17 μm, and after excluding guard zones ∼15 μm, resulted in a hsf of 0.21. Each microglia cell was scored as either “non-activated,” “intermediate” or “phagocytic” based on the following criteria: phagocytic cells were completely amoeboid in nature, with no processes extending from the cell body; non-activated microglia were single cells with small soma and long processes extending away from the cell body; intermediate cells included rod-shaped microglia and any cell that did not meet the other two criteria. The average number of sample sites, cells counted, estimated cell counts, and coefficient of error (CE) are reported in Table 2.

**Table 2:** Sampling parameters for stereology counts of IBA-1 cells in the hippocampus following TBI.

	Sham	TBI
**Sample Size** (# animals)	5	5
**Average Sample Sites [Range]**	185 [173–195]	181 [175–186]
**Average Cells Counted [Range]**	344 [304–372]	908 [276–1274]
**Average Est. Cell Count [Range]**	20,421 [7875–30,738]	49,908 [20,738–78,913]
**Coefficient of Error (CE) [Range]**	0.083 [0.076–0.087]	0.066 [0.047–0.094]

High-content images for automated assessment of microglial activation following TBI were collected and analyzed via the automated algorithm as described above. Automated morphology ratios were correlated with the stereological classifications listed above, e.g., “phagocytic,” “intermediate,” “non-activated.” Metrics to evaluate microglia cell activation included total cell number, morphology ratio, and percent area.

### 2.7 Statistical analyses

Analyses were split into validation and exploratory cohorts. For the exploratory cohort, outcomes included each of the parameters generated by the algorithm, measured across cells (or cell clusters), brain slices (2–3 per animal), and animals. Based on preliminary analyses, number of cells, area of cell body, area of cell, process length, ratio of process length and cell body area, CD68 intensity, perimeter, and number of endpoints of cell processes were transformed using the natural logarithm to better meet the model assumption of constant variance; due to some zeros in the data, the number of endpoints was first shifted by 0.5 prior to taking the natural logarithm. Repeated measures, random effects models were used to estimate differences between groups (activated, non-activated, vehicle). Contrasts were constructed for the pairwise comparison of each group. Models also included an animal-specific random intercept to account for the repeated measurements across cells/cell clusters identified within brain slices of an animal. Model assumptions were assessed and met by the data. Due to extreme outliers, models for log cell body area and log process length were restricted to cell clusters of size 15 or smaller (including single cells). For log-transformed outcomes, differences are presented as geometric mean ratios (GMRs), which may be interpreted as fold changes, so that a ratio of 1.5 corresponds to a 50% increase and a ratio of 0.5 corresponds to a 50% decrease. A further analysis compared parameters generated by the algorithm across sham or TBI animals. All parameters generated by the algorithm, except CD68 intensity, were used as outcomes. The analyses paralleled those described above for the exploratory cohort. For all other outcomes, differences are expressed on the original scale. All analyses were conducted using SAS, version 9.4 and a p-value less than 0.05 was considered statistically significant. For the validation of the automated algorithm, measures of morphology ratio, a Spearman correlation coefficient and its 95% confidence interval (CI) were computed. The estimated correlation within each animal was also evaluated.

All other analyses were conducted using GraphPad Prism version 8.1.2. Comparisons of microglia cell counts between DFP and VEH, or TBI and sham animals were analyzed using Mann-Whitney tests. Spearman correlations determined the strength of the relationship between the automated morphology ratio generated by the algorithm and the score given by a trained investigator, as well as the comparison between the cell counts obtained manually and by the algorithm. Additionally, Spearman correlations examined the relationship between stereology counts and automated counts of IBA-1 cells in TBI and sham animals. Automated morphology ratios for IBA-1 cells in TBI and sham animals were analyzed using mixed-effects models with Sidak's multiple comparisons. Lastly, a two-way ANOVA with Sidak's multiple comparisons determined statistically significant differences between TBI and sham animals for phagocytic, intermediate, or non-activated populations of IBA-1 cells.

## 3 Results

### 3.1 Manual assessment confirms robust microglial activation in the rat DFP model

**Fig. 1:**
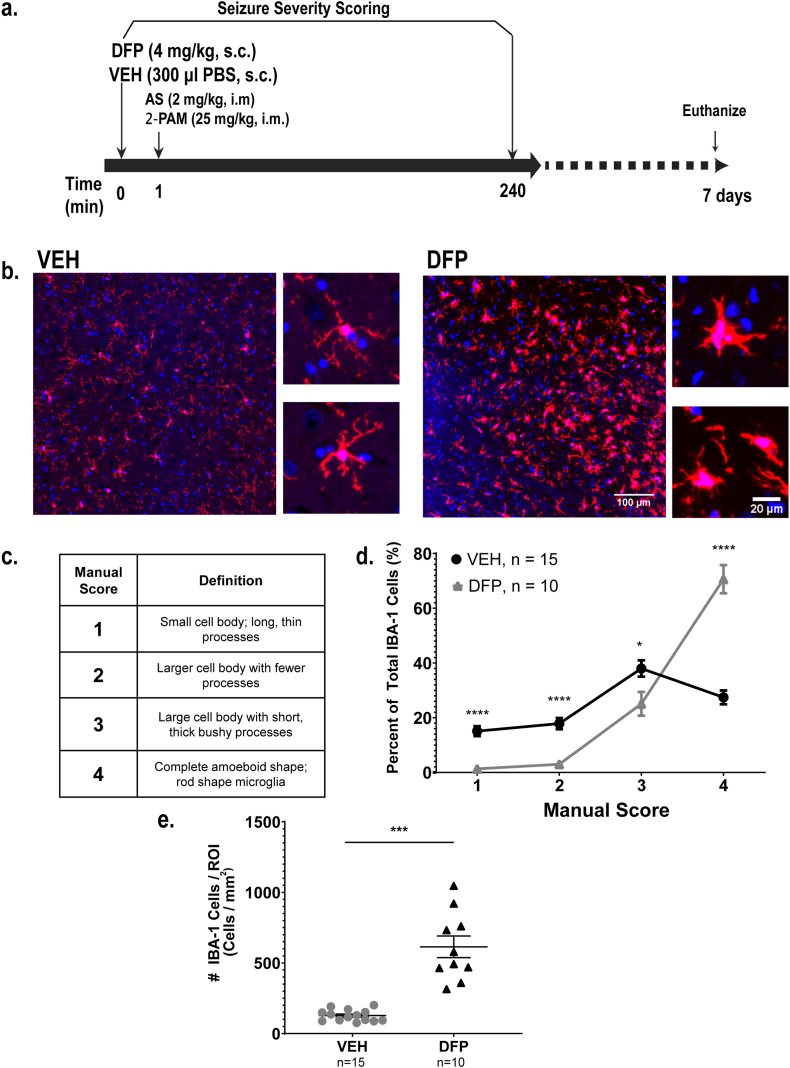
Manual assessment of microglial morphology in the dorsal lateral thalamus of vehicle (VEH) vs. DFP intoxicated animals at 7 days post-exposure (DPE). **a.** Schematic illustrating the exposure paradigm. Animals were euthanized at 7 DPE and brains perfused and then post-fixed for immunohistochemistry. **b.** Representative photomicrographs of DAPI (blue) staining and IBA-1 immunolabeling (red) in the dorsal lateral thalamus of VEH and DFP animals. Scale bar = 100 μm (inset = 20 μm). **c.** Criteria used to manually score microglial morphology in rat brains. **d.** Quantitative assessment of microglial morphology scored manually in the exploratory data set. Data presented as the mean ± S.E. (n = 15 VEH animals and 10 DFP animals). Significantly different from VEH at *p < 0.05, ****p < 0.00001 as determined by *t*-test. **e.** Quantification of the number of IBA-1 cells per ROI (#IBA-1 Cells/mm^2^). Data presented as mean ± SE with each data point representing a single animal (n = 15 VEH animals and 10 DFP animals). ***Significant at p < 0.001 as determined by Mann-Whitney test.

Microglial activation is a known consequence of OP-induced *status epilepticus* (SE) and has been assessed using a variety of different methods [39,40,47]. Here, we confirm that microglial morphology in the dorsal lateral thalamus is significantly altered 7 days after acute DFP intoxication compared to vehicle (VEH) controls as evaluated by IBA-1 immunoreactivity (Fig. 1). Specifically, in the dorsal lateral thalamus of animals exposed to DFP, the population of microglia with amoeboid morphology was found to be significantly higher than in vehicle (VEH) controls when manually scored on a 4-point scale by an investigator blinded to all experimental groups (Fig. 1d; mean: VEH 2.8; DFP 3.6). In addition, DFP animals had significantly increased numbers of IBA-1 immunopositive cells per unit area relative to VEH animals (Fig. 1e; mean ± SD, VEH: 127 ± 40.74; DFP: 614.1 ± 242.8), confirming that acute DFP-intoxication causes robust changes in microglial morphology at 7 DPE.

### 3.2 Automated analysis algorithm for unbiased quantification and classification of fluorescently labeled IBA-1 immunoreactive microglia

**Fig. 2:**
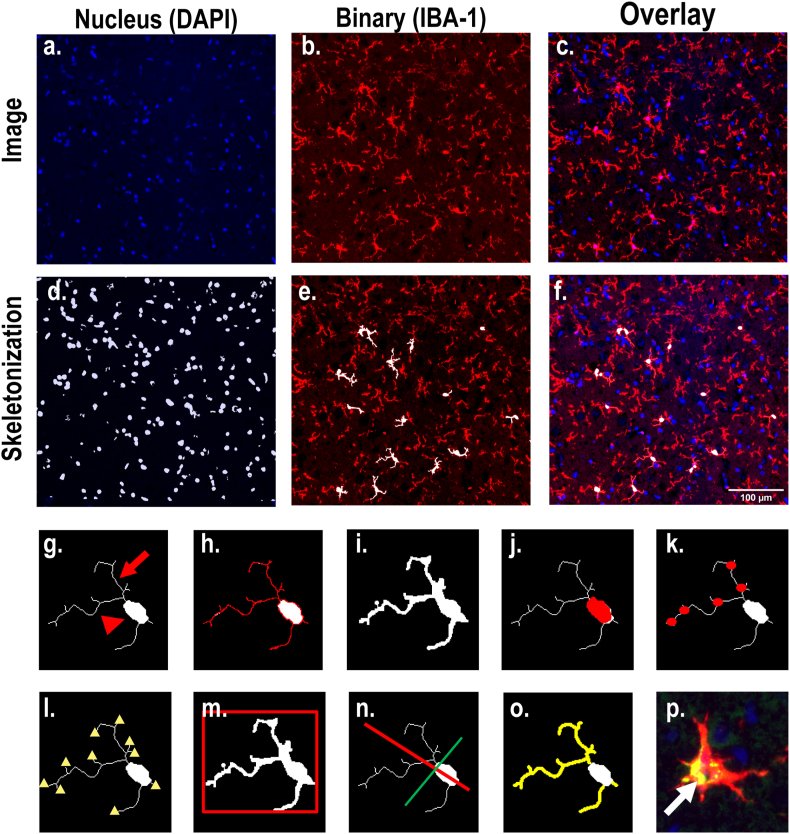
Automated image analysis pipeline for fluorescent images of the dorsolateral thalamus and description of parameters to define microglial activation. **a-c.** Photomicrographs of **(a)** DAPI **(b)** IBA-1 and an **(c)** overlay of the two channels. ***d*-f.** Examples of superimposed skeletonization for **(d)** DAPI nucleus watershed **(e)** IBA-1 binarization, in which only cells containing a soma and a nucleus are selected to avoid analyzing cell fragments from other layers and **(f)** oligo overlay. Scale bar = 100 μm. **g-p.** Binary skeletonization of a single microglia depicting **(g)** automated morphology ratio, which is the process length (arrow) divided by the cell body area (arrowhead); **(h)** total length of cell perimeter (red); **(i)** total area of the cell without skeletonizing the processes; **(j)** area of cell body (red); **(k)** number of branch points within line structures (red dots); **(l)** number of endpoints within line structures (yellow triangles); **(m)** percent fill, which is the ratio of the binarized cell area to a rectangle (red) with dimensions to fit the entire cell; **(n)** span ratio of the major axis (red) to the minor axis (green) of the cell; **(o)** total length of the processes of the cell (yellow); and **(p)** total intensity of CD68 within the area of the IBA-1 binary mask (white arrow). MATLAB scripts used for analysis, statistics and visualization can be found under https://github.com/Lein-Lab/microglia-phenotyping.

To overcome key barriers to manual quantification of microglial morphology, including a limited 4-point semi-quantitative scale, the inherent susceptibility to bias, as well as time and labor demands, we developed an algorithm (available at https://github.com/Lein-Lab/microglia-phenotyping) that rapidly quantifies microglial morphology, analyzing every cell in the region of interest with high sensitivity and low bias. A written protocol outlining all steps required to run this algorithm is provided in the Appendix. The first step of this algorithm was to convert the fluorescent images into binary masks for individual channels (Fig. 2d and e) before combining them into a final skeleton that defines the cell soma and separate it from the processes (Fig. 2f). Each binarized image was then analyzed for 10 different metrics that define microglial activation. Nine metrics are based upon the shape of the cell (Fig. 2g - o) and the tenth assesses phagocytic activity by quantifying expression of CD68, a biomarker of phagocytic activity in microglia (Fig. 2p). Metrics that were used to define the cell shape included a morphology ratio (Fig. 2g), which is the ratio of cell process length to cell body area (a larger morphology ratio indicates a more resting-like cell); perimeter of the cell (Fig. 2h); total area of the cell after applying the binary mask (Fig. 2i); cell body area (Fig. 2j); number of branch points per cell (Fig. 2k); number of process tips per cell (Fig. 2l); percent fill or the ratio of binarized cell area to a rectangle (red) with dimensions to fit the entire cell (Fig. 2m); span ratio (Fig. 2n), which is the ratio of the major axis to the minor axis of the cell; total length of the cell processes (Fig. 2o); and the total intensity of CD68 within the area of the cell mask (Fig. 2p). Of note, this method is not limited to CD68. This code (freely available and modifiable) can be adapted to include a fluorescent marker for any target antigen, or even multiple fluorescent markers, for any cell type (e.g., GFAP for astrocytes). However, additional background corrections will need to be applied for each fluorescent biomarker depending on the signal to noise ratio.

**Fig. 3:**
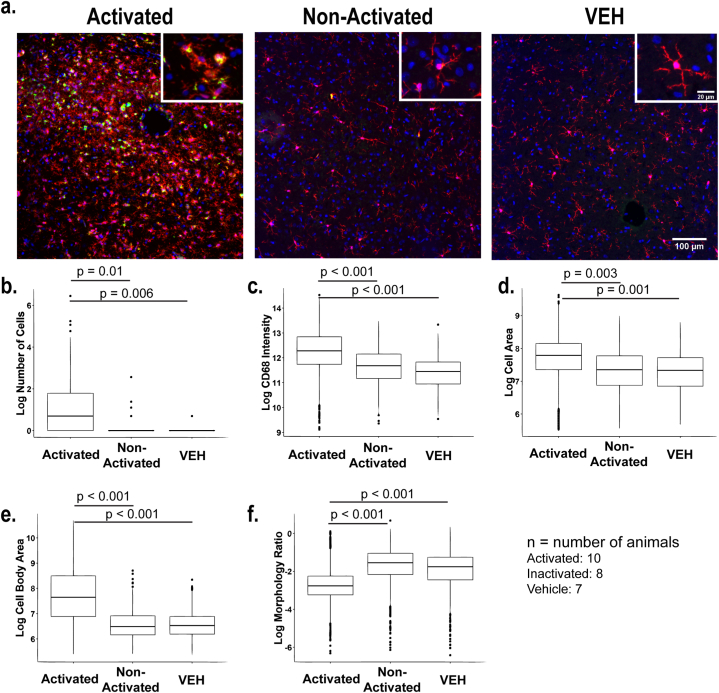
Automated algorithm identifies eight sensitive metrics for detecting changes in microglial activation states in exploratory data set. **a.** Representative photomicrographs of IBA-1 (red), CD68 (green), and DAPI (blue) immunolabeling in the dorsal lateral thalamus of three different populations of microglia: activated, non-activated, and vehicle control animals. Activated populations were selected based on amoeboid microglia that were CD68 positive. Scale bar = 100 μm (inset = 20 μm). **b-f.** To meet the assumption of constant variance, all values were log-transformed, and quantification of the log transformed values are plotted for **(b)** number of cells, **(c)** total CD68 intensity, **(d)** cell area, **(e)** cell body area and **(f)** morphology ratio in activated vs. non-activated vs. VEH groups. Statistical significance was determined at p < 0.05 as determined by repeated measures, random effect models and is denoted for each pairwise comparison. N represents number of animals for each group.

The algorithm was developed using images from the dorsal lateral thalamus that were generated from 18 of the 25 animals in the study (n, VEH: 8; DFP: 10). Because the investigator who generated these images was blinded to experimental group, images were initially separated into two groups based on whether the majority of cells appeared to be in an “activated” or “non-activated” state (Fig. 3a) as determined by 1) the morphology of cells; 2) total number of cells within a frame; 3) number of cells that clustered together (Fig. 3a inset); and 4) total expression of CD68 within the image. Both the “activated” and “non-activated” groups were then compared to a group of 7 VEH animals to validate the method (Fig. 3a).

The algorithm determined morphological parameters for an average of 451 IBA-1 immunopositive cells or cell clusters per animal (range 79 - 1456), with 56% consisting of single cells and an additional 30% being cell clusters with an estimated 2–15 cells per cluster (range of cell clusters: 2–632). Cell number differed between groups (p < 0.001), with a greater number of cells present in the activated group relative to both the non-activated group (p = 0.01) and the VEH group (p = 0.006) (Fig. 3b). Results were confirmed when restricted to clusters of 15 cells or less. For subsequent analyses, cell clusters with greater than 15 cells were excluded from the data set to control for extreme outliers. Analyses of other parameters included cell number as a covariate due to this difference.

The most sensitive parameters for detecting significant differences between groups included CD68 fluorescence intensity, total cell area, cell body area, morphology ratio, and number of branching points per cell (Table 3). Activated cells exhibited CD68 intensity values twice that of VEH cells and 55% higher than non-activated cells (Fig. 3c). Total cell area was 36% higher in activated cells than VEH and 32% higher than non-activated cells (Fig. 3d). Activated cells also had a 95% higher cell body area than VEH and twice the cell body area than non-activated cells (Fig. 3e). The morphology ratio was 46% lower in activated cells than VEH and 56% lower in activated cells than non-activated cells (Fig. 3f). The number of branching points was lower in activated cells than in VEH and non-activated cells, such that, on average, activated cells had 1.2 fewer branching points than VEH and 1.5 fewer branching points than inactivated cells.

**Table 3:** Summary of statistical values for outcomes that define microglial activation states in fluorescent images.

Parameter	Activated vs. Inactivated			Activated vs. VEH		
	Difference	95% CI	Significance	Difference	95% CI	Significance
**Morphology Ratio**	GMR = 0.44	0.32, 0.61	p < 0.001	GMR = 0.54	0.39, 0.75	p < 0.001
**Perimeter**	p = 0.8					
**Area of Cell**	GMR = 1.32	1.10, 1.60	p = 0.003	GMR = 1.36	1.13, 1.65	p = 0.001
**Area of Cell Body**	GMR = 2.04	1.55, 2.67	p < 0.001	GMR = 1.95	1.48, 2.56	p < 0.001
**Number of Branching Points**	−1.5	−2.53, −0.48	p = 0.004	−1.2	−2.23, 0.17	p = 0.02
**Number of Endpoints**	GMR = 1.18	1.02, 1.37	p = 0.03	GMR = 1.18	1.01, 1.37	p = 0.04
**Percent Area**	0.03	0.009, 0.06	p = 0.008	0.008	−0.02, 0.03	p = 0.5
**Span Ratio**	−0.14	−0.26, −0.02	p = 0.02	−0.1	−0.22, 0.02	p = 0.1
**Process Length**	p = 0.7					
**CD68 Intensity**	GMR = 1.55	1.13, 2.13	p = 0.007	GMR = 2.02	1.46, 2.77	p < 0.001

GMR = geometric mean ratio of value for activated vs. non-activated or for activated vs. VEH groups. All other values are listed on the original scale. Green highlighted values represent significance as determined by repeated measures, random effects models.

Other parameters that indicated significant differences between groups included percent area and span ratio (Table 3). Percent area was slightly higher in activated cells than non-activated cells, but not VEH. Span ratio was lower in activated cells than non-activated cells, but not VEH. The number of endpoints were not statistically different between groups (p = 0.07), although when restricted to cell clusters of size 15 cells or fewer to eliminate any outliers in the data, activated cells had a higher number of endpoints than VEH and non-activated cells. There were no differences between groups in process length (p = 0.7) or perimeter (p = 0.8).

### 3.3 Validation of the algorithm demonstrates strong correlation between automated and manual scoring

**Fig. 4:**
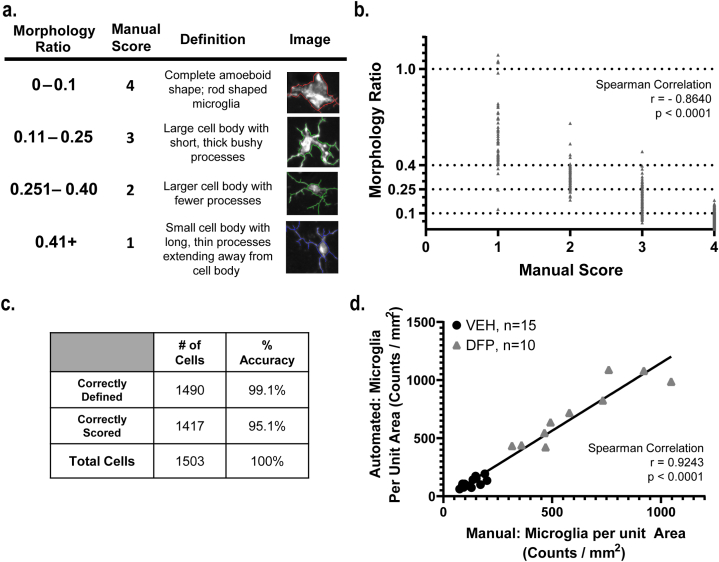
Manual validation is strongly correlated with automated analysis platform in fluorescent validation data set. **a.** Comparison of the automated morphology ratio, manual score, description of manual scoring criteria and representative images for each category. **b.** Graphical representation of manual scoring vs. automated morphology ratio in all images within validation data set. Dotted lines crossing the y-axis represent the cut-off values for the automated morphology ratios. Spearman correlation: r = −0.8640, 95% CI: (−0.8767, −0.8502), p < 0.0001. **c.** The accuracy of the algorithm in defining and scoring microglial cells. **d.** Graphical representation of manual vs. automated scoring of the number of microglia counted per unit area for each animal. Spearman correlation: r = 0.9243; 95% CI: 0.8267 to 0.9679; p < 0.0001.

An additional data set was analyzed using the validation mode of MeasureMicroGliaActivation to manually assess the morphology ratio of random samples of individual cells. Each cell image was scored on a semi-quantitative scale [50] (Fig. 4a) by an investigator blinded to experimental group. The validation set consisted of data from 21 animals, and the number of cells analyzed ranged from 25 to 167 per animal. The automated morphology ratios were significantly correlated to manual scores within each animal, and all but six animals had correlations ranging from −0.96 to −0.50, which were of similar magnitude to the pooled data set (Fig. 4b).

The accuracy of the algorithm was assessed based on two criteria: 1) ability to correctly identify the soma & processes of a single cell, and 2) assignment of a morphology ratio that corresponded to the manual score (Fig. 4a). The algorithm was incorrect when it identified a cell that the investigator could not identify by eye or when the morphology ratio was outside the corresponding range for manual assessment. It is important to note that accuracy in this case refers to the correlation between the manual assessment and the automated morphology ratio, not the accuracy of tracing microglia processes. The latter does not have a dramatic impact on outcome because thousands of cells were analyzed – tracing accuracy would be a much bigger concern if fewer cells were studied at higher magnification. Approximately 1503 cells were identified by the algorithm (1.4% false negative rate, n = 21 cells) with over 99% (n = 1490) being correctly identified as true positives (0.86% false positive rate: n = 13 cells) and of those cells, 95.1% (n = 1417) were scored the same as a trained investigator (4.8% false negative rate, n = 73 cells; Fig. 4c). The cell counts generated by the algorithm were compared back to the counts assessed manually for each animal (Fig. 4d) and found to strongly correlate with manual cell counts (Fig. 4d, rho = 0.9243, 95% CI: (0.8267–0.9679), p < 0.001).

### 3.4 The automated algorithm quantifies morphometric criteria of cells labeled via chromogenic methods

**Fig. 5:**
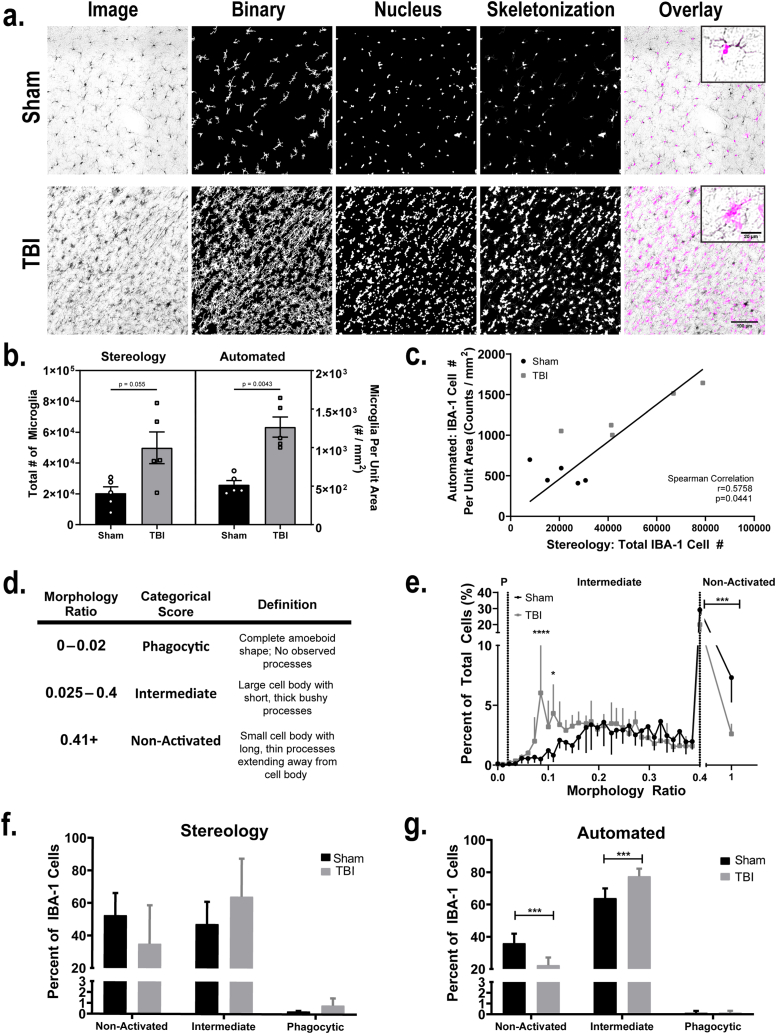
Automated algorithm detects changes in microglial morphology following traumatic brain injury (TBI) in chromogen labeled tissue. **a.** Representative photomicrographs of the CA3 sub-region of the hippocampus in sham (top) and TBI animals (bottom) immunostained for IBA-1. The column (from left to right) represents an image uploaded to the algorithm software, binary skeletonization of IBA-1 immunolabeling in the image, nucleus watershed, oligo skeletonization of cell body vs. processes and overlay of the oligo skeletonization with the photomicrograph. Scale bar = 100 μm (inset = 20 μm). **b.** Estimation of total IBA-1 cells determined by stereology (left) and the automatic quantification of IBA-1 cells (right) in sham (black) and TBI (gray) animals. Data presented as mean ± SEM. Significantly different at p < 0.05 as determined by Mann-Whitney test. **c.** Relationship between the total numbers of IBA-1 cells counted via stereology methods (x-axis) and automated methods (y-axis). Black dots represent sham animals and gray dots represent TBI animals. **d.** Table summary describing the automated morphology ratio, categorical stereology score, and the description of the scoring criteria used. **e**. Distribution of automated morphology ratios for each animal in sham (black plots; n = 5) and TBI groups (gray plots; n = 5). Data presented as mean ± SD. Dotted lines that cross the x axis represent cutoff morphology ratios for phagocytic (P), intermediate, and non-activated categorical scores. Significantly different from sham at *p < 0.05; ****p < 0.001 as determined by mixed-effects models with Sidak's multiple comparisons. **f-g.** Total percentage of IBA-1 cells for each categorical assignment in sham (black) and TBI (gray) animals as determined by stereology counting methods (**f**) or automated methods (**g**). Data presented as mean ± SD, n represents the same animals represented in (**e**). Significantly different from sham at ***p < 0.001 as determined by two-way ANOVA with Sidak's multiple comparisons.

To determine whether the algorithm could be used with methods other than fluorescent images, we generated a data set consisting of chromogenic labeled IBA-1 cells from a study of microglia in the hippocampus of sham vs. traumatic brain-injured rats. Consistent with the literature [41,42], we saw robust increases in microglial activation within the CA3 region of the hippocampus 14 days following TBI (Fig. 5a). Data were generated using the automated algorithm from a total of 9539 cells from 11 animals (sham: 5; TBI: 5) using 1–2 brain sections per animal. CD68 immunoreactivity was removed as a parameter as sections were only immunolabeled for IBA-1. (Fig. 2d-l). The algorithm generated morphology parameters for an average of 867 cells or cell clusters per animal (range 332 - 1506), with 35% consisting of only one cell and 62.4% having an estimated 2–15 cells within the cluster (range 1 - 25). Animals with TBI had more cells in a cluster than sham animals (GMR = 1.48, 95% CI: (1.03, 2.13), p = 0.03). The results were similar when statistical analysis was restricted to cell clusters of 15 cells or less. Therefore, statistical analyses of additional parameters included cell number as a covariate in the model.

The greatest differences between TBI and sham animals included morphology ratio, percent area, and perimeter (Table 4). The morphology ratio was 20% lower in TBI animals than sham animals (GMR = 0.80, 95% CI: (0.68, 0.96)), and the percent area was 0.05 units (5% points) larger in TBI animals than shams (difference = 0.05, 95% CI: (0.03, 0.08), p < 0.001). The perimeter was also 19% lower in TBI animals than sham animals (GMR = 0.81, 95% CI: (0.70, 0.94)). The number of branching points in TBI animals was not significantly lower than sham animals (difference = −0.5, 95% CI: (−1.0, 0.03), p = 0.06)) and there was no difference between the two groups of animals for cell area (p = 0.09), area of the cell body (p = 0.9), span ratio (p = 0.99), number of endpoints (p = 0.3), or process length (p = 0.2).

**Table 4:** Summary of statistical values for outcomes that define microglial activation states in chromogenic images.

Parameter	TBI vs. Sham		
	Difference	95% CI	Significance
**Morphology Ratio**	GMR = 0.8	0.68, 0.96	p < 0.001
**Perimeter**	GMR 0.81	0.70, 0.94	p < 0.001
**Area of Cell**	p = 0.09		
**Area of Cell Body**	p = 0.90		
**Number of Branching Points**	−0.5	−1.0, 0.03	p = 0.06
**Number of Endpoints**	p = 0.3		
**Percent Area**	0.05	0.03, 0.08	p < 0.001
**Span Ratio**	p = 0.99		
**Process Length**	p = 0.2		

GMR = geometric mean ratio of value for TBI vs Sham groups. All other values are listed on the original scale. Green highlighted values represent significance as determined by repeated measures, random effects models.

### 3.5 Comparison of the automated algorithm to stereological analyses

In our initial analysis with the automated algorithm, we only captured 1–2 brain sections per animal. We wanted to determine how sampling of IBA-1 cell numbers with the automated algorithm compared with traditional stereological analysis of brains sections from the same animals. Sample sizes, number of sampling sites and CE values for stereology data can be found in Table 2. Both stereology and automated counts determined that TBI animals had greater numbers of IBA-1 cells than sham animals [Fig. 5b, Stereology mean ± SD: TBI: 49,908 ± 23,019 cells; sham: 20,421 ± 9273 cells; Automated mean ± SD: TBI: 1267 ± 292 cells/mm^2^; sham: 502.1 ± 123 cells/mm^2^]. Based on the sampling methods used in this study, the automated algorithm was not able to provide volumetric counts for the total number of IBA-1 cells. However, within an analyzed region, every IBA-1 positive cell was counted. When comparing the volumetric counts of stereology to the counts provided by the automated algorithm in the same animals, there is a strong linear relationship between the two methods (Fig. 5c, rho = 0.58, p = 0.0441). These data suggest that while stereology provides the best estimate of the total numbers of cells within a region, the automated algorithm can accurately determine relative differences between experimental groups.

With stereology, there is significant risk for introducing investigator bias, morphometric data are often semi-quantitative or categorical in nature, and there is the risk for decreased sensitivity. We compared the continuous nature of automated morphology ratios generated by the automated algorithm to categorical scores used by investigators to evaluate microglial profiles in the TBI study samples (Fig. 5d). Scoring using stereology determined that TBI animals trended towards having greater percentages of phagocytic or intermediate microglia and lower percentages of non-activated microglia compared to sham animals; however, these differences were not statistically significant (Fig. 5f). Alternatively, when comparing morphological profiles using the continuous scale provided by the automated algorithm, TBI animals had significantly greater numbers of intermediate microglia (p = 0.0002) but lower numbers of non-activated microglia compared to sham controls (p = 0.0002, Fig. 5g). Furthermore, due to its use of a continuous scale, the automated algorithm was better able to detect the degree by which microglial morphology changes across a continuum (Fig. 5e). Neither method detected any differences in phagocytic microglia between TBI and sham animals.

### 3.6 Discussion

We developed a fully automated image analysis tool that is both highly sensitive and accurate in detecting and quantifying complex morphometric changes in microglial cells. Additionally, this method combines quantitative metrics of cell morphology with expression of immunocytochemical biomarkers (e.g., CD68 in our studies) to rapidly screen for biological changes in large populations of cells. Importantly, analysis of both fluorescent and chromogenic images highlights the versatility of this tool compared to previously published methods. To validate the development of this tool, we took advantage of well-established preclinical rat models that exhibit robust microglial responses: a rat model of acute intoxication with DFP [39,40,47], and a rat model of experimental TBI [41,51]. While each model is unique with regard to the spatiotemporal progression of microglial activation in the brain, the morphometric features of microglia as detected using immunohistochemical methods are similar in both models. Importantly, while this tool was developed using microglia morphologies, the algorithm can be easily adapted by investigators to evaluate the cytoarchitecture of any cell type and any fluorescent or chromogenic label.

The automated algorithm analyzed ten parameters that describe microglial phenotypes: nine relating to morphological features and one defining a protein biomarker of activation (e.g., CD68). When comparing “activated” and “non-activated” populations of microglia in DFP intoxicated animals, we found that 8 of the 10 parameters were statistically significant between these populations; however, only 6 of the 10 were significant when we compared activated vs. VEH populations. Percent area and span ratio were the two parameters that dropped out in the latter comparison, likely as treatment-related changes were not robust enough for multiple group comparisons. Surprisingly, total process length and the perimeter of the cell were not found to be significant metrics in either comparison; however, upon further examination of cell morphology, we realized that the transition from a ramified structure to amoeboid shape is a continuous process, and microglia have some processes extending away from the cell body until reaching a terminal amoeboid shape (Fig. 2). This observation was confirmed by “activated” microglia exhibiting a greater number of endpoints than “non-activated” or VEH cells (Table 3). Collectively, these analyses confirm the sensitivity of this algorithm to capture changes in microglial morphology across a continuous spectrum. As cells transition towards being fully amoeboid, they retract their processes while losing branching complexity, increasing cell body area and increasing expression of CD68 [52].

We acknowledge ours is not the first automated tool for assessing morphometric parameters of microglia [31,32,34,36,38]; however, we have expanded on other open-source methods by increasing the throughput of single-cell analysis (e.g., <30 min to identify and quantify ∼500 cells vs. hours to days for existing methods [35,38]) introducing the capability for both fluorescent and chromogenic detection methods, as well as incorporating additional biomarkers of activation. For example, our automated algorithm can provide information about phagocytic activity when multiple fluorophores are used by examining the intensity of the biomarker CD68 in addition to IBA-1 within each cell. CD68 is a member of the lysosomal/endosomal-associated membrane glycoprotein (LAMP) family. Increased CD68 expression suggests increased phagocytic activity of monocytes and tissue macrophages [53]. When co-expressed with IBA-1 within the brain, this is likely indicative of increased phagocytic activity of microglia or invading myeloid cells in the brain parenchyma. We know that the number of CD68 immunopositive cells increases significantly in the brain of animals acutely intoxicated with DFP compared to VEH controls [47], and this proved true here when our automated method assessed the activation status of microglia.

A major advantage of our algorithm is that it’s not limited to biomarkers of microglial activation but can be adapted and optimized to evaluate diverse biomarkers of microglia and of different cell-types, e.g., GFAP for astrocytes or SOX10 for oligodendrocytes [54,55]. Importantly, while we have optimized the platform for indirect immunofluorescence detection methods, e.g., immunohistochemistry, it is entirely possible to extend this platform for use with direct fluorescence, e.g., transgenic animals with cell-type-specific fluorescent promoters – possibly improving the signal-to-noise ratio of the data [56]; however, background corrections would need to be adjusted for each fluorescent marker. Finally, the versatility of this system is demonstrated by its accurate detection of changes in microglial morphology in brain tissues processed using chromogenic methods. While fluorescence can have high signal-to-noise and allows for multiple antibodies to be used, chromogenic methods are straightforward, cost-effective, and the antigen-antibody complex can be visualized using standard microscopy techniques [20,22,57]. The versatility of our system gives investigators the flexibility to choose either fluorescent or chromogenic methods and specific biomarkers based on the experimental and economical needs of their study.

Importantly, our fully automated algorithm not only increases throughput for analyzing individual cells compared to other published methods, but also removes the need for any investigator interpretation – limiting unintentional bias within a data set. Existing methods have been designed for high-magnification images of pre-selected cells, which is important to accurately trace cell processes in 3D z-stacks, however, this significantly limits the throughput of single-cell analysis and introduces investigator bias in cell selection [32,34,36,38]. By coupling our analysis software with high-content imaging, we can quickly screen for quantitative changes in 8 morphological characteristics of single cells within entire brain regions in a 2D section, thereby eliminating any investigator bias. While not explicitly examined in this study, it seems likely that this platform could be readily adapted for high-throughput screening of cultured cells.

A significant goal in analyzing large imaging data sets is to balance rapid throughput with scientific rigor and reproducibility. To address this issue, we performed a side-by-side comparison of our automated analysis platform with traditional stereological methods in chromogen-labeled brain tissues following TBI. Traditionally, stereology is used to estimate cell numbers in a three-dimensional structure. It is widely considered to be the most rigorous standard for providing the closest approximation of absolute cell counts over a three dimensional volume [58]. One common application for stereological quantification is to determine absolute numbers of cells within a particular region of interest by randomly and systematically sampling a relatively few number of cells over the entire volume of that region [59]. Using this method, we found, as expected, that TBI animals had increased numbers of IBA-1 immunopositive cells [41]. In contrast to stereology, the automated algorithm captures every single cell within each section. When we compared the numbers of microglia obtained using stereology vs. the automated algorithm in the same brain sections, we found that the automated algorithm does not provide the same approximation of absolute cell numbers as stereology, not surprising given that only 2 brain sections were sampled using the automated algorithm. However, the counts generated by stereology vs. the automated algorithm are highly correlated, suggesting that the automated algorithm can provide a similar relative measure of cell counts and may be used as a good screening tool for such purposes. If there is concern about sample bias in a specific application, for example, a variable pattern of activation around a lesion, there is no limit to the number of sections that can be included to improve rigor using high content imaging. In future studies, it will be necessary to determine whether quantification of additional sections, for example sampling every fifth section similar to our stereological counts, using the automated algorithm can provide estimates of the total number of microglia that are closer to the absolute cell count obtained using stereology. If investigators were to use high content imaging to capture the entire region of interest, we believe that assessment of the total number of microglia should be consistent between stereology and our automate algorithm.

While stereology often gives the most reliable estimates of absolute cell counts, a major limitation arises when it is used to assess cellular morphology because this type of histological analyses is often categorical in nature and has a high likelihood of introducing investigator biases when assigning a particular score [60]. A significant advantage of our automated algorithm relative to stereology is that the former provides an unbiased assessment based on a continuum of morphologies that integrates analysis of 8 structural parameters. Investigator bias in categorizing morphological phenotypes can make it challenging to reproduce particular data sets, especially when different investigators score the same data set [60]. However, automated morphological analysis with our automated algorithm results in single cells being scored on a continuous scale and subtle morphometric differences that may have been missed when using categorical assignments are identified. This was evident with the TBI data set. While stereology detected that TBI animals trended towards having more phagocytic and intermediate microglia compared to sham controls, there were no statistical differences between the groups. However, analysis of the same images using the automated algorithm detected significantly greater percentages of IBA-1 cells with an intermediate activation stage. Neither method uncovered a change in the percentage of the total population represented by this phenotypic category as determined using predetermined scoring criteria.

When choosing tools to analyze morphological phenotypes [32,34,38], it is important to consider the central question or hypothesis of the study. For example, if one expects an experimental manipulation to cause subtle changes in cell numbers, stereology may be the more sensitive method [59]. On the other hand, if one is concerned about morphological phenotypes, such as the morphological profile of microglia, the continuous nature of the automated algorithm may provide a more reliable and sensitive analysis. Time for analysis is another factor to consider; the automated algorithm is extremely efficient and can analyze thousands of cells in a matter of hours. An investigator could use the algorithm as a screening tool to identify treatments or experimental manipulations to be further analyzed using more time-intensive methodologies, such as stereology. In addition, the ability of the automated algorithm to quickly image thousands of cells at a time reduces the extent of photobleaching that would otherwise occur with fluorescent antibody detection performed using traditional stereology on a microscope [61]. Finally, by reducing the time necessary to image (high content) and count (algorithm), investigators may be encouraged to evaluate multiple regions of interest across the brain rather than focusing on just a few brain regions.

In summary, we have developed a novel algorithm that advances quantitative analyses of cellular morphology by automating the quantification of cytoarchitecture in an unbiased and higher-throughput manner compared to existing methods and by enabling integration of structural analyses with quantitative analyses of immunocytochemical biomarkers. Furthermore, our freely available analysis pipeline offers a novel and customizable approach for analyzing both morphological and protein expression that investigators can extend to other cell types with complex and dynamic morphologies. This technique provides a powerful tool for identifying quantitative, dynamic changes in cell morphology that can be used to rapidly screen for changes in cellular features in diverse models.

## Author contribution statement

Michelle Guignet; Martin Schmuck: Conceived and designed the experiments; Performed the experiments; Analyzed and interpreted the data; Wrote the paper.

Danielle J. Harvey: Analyzed and interpreted the data; Contributed reagents, materials, analysis tools or data; Wrote the paper.

Danh Nguyen; Gene Gurkoff: Analyzed and interpreted the data; Contributed reagents, materials, analysis tools or data.

Donald Bruun: Contributed reagents, materials, analysis tools or data.

Angela Echeverri: Performed the experiments.

Pamela J Lein, Ph.D.: Conceived and designed the experiments; Analyzed and interpreted the data; Contributed reagents, materials, analysis tools or data; Wrote the paper.

## Funding statement

Dr. Pamela J Lein was supported by 10.13039/100000065National Institute of Neurological Disorders and Stroke [U54 NS079202], 10.13039/100009633Eunice Kennedy Shriver National Institute of Child Health and Human Development [P50 HD103526].

## Data availability statement

Data will be made available on request.

## Declaration of interest's statement

The authors declare that they have no known competing financial interests or personal relationships that could have appeared to influence the work reported in this paper.

## CRediT author statement

**Michelle Guignet:** Conceptualization, Validation, Investigation, Data Curation, Writing – Original Draft, Visualization. **Martin Schmuck:** Conceptualization, Methodology, Software, Validation, Formal Analysis, Data Curation, Writing – Original Draft, Visualization. **Danielle Harvey:** Methodology, Formal Analysis, Data Curation, Writing – Original Draft, Writing – Review & Editing. **Danh Nguyen:** Methodology, Formal Analysis, Data Curation, Writing – Original Draft, Writing – Review & Editing. **Donald Bruun:** Resources. **Angela Echeverri:** Investigation, Resources. **Gene Gurkoff:** Methodology, Resources, Writing – Original Draft, Writing – Review & Editing, Supervision. **Pamela Lein:** Conceptualization, Resources, Writing – Review & Editing, Supervision, Project Administration, Funding Acquisition.
